# Mechanical and Fire Properties of Flame-Retardant Laminated Bamboo Lumber Glued with Phenol Formaldehyde and Melamine Urea Formaldehyde Adhesives

**DOI:** 10.3390/polym16060781

**Published:** 2024-03-12

**Authors:** Ying He, Xiaobei Jin, Jingpeng Li, Daochun Qin

**Affiliations:** 1 Department of Biomaterials, International Centre for Bamboo and Rattan, Beijing 100102, China; heying@icbr.ac.cn; 2 SFA and Beijing Co-Built Key Laboratory of Bamboo and Rattan Science & Technology, State Forestry, Administration, Beijing 100102, China; 3 Key Laboratory of High Efficient Processing of Bamboo of Zhejiang Province, China National Bamboo Research Center, Hangzhou 310012, China; 4 Sanya Research Base, International Centre for Bamboo and Rattan, Sanya 572000, China; qindc@icbr.ac.cn

**Keywords:** laminated bamboo lumber, mechanical and fire properties, flame-retardant treatment, PF and MUF adhesives

## Abstract

This study investigated the effects of different adhesives, phenol formaldehyde (PF) and melamine urea formaldehyde (MUF), on the mechanical and fire properties of flame-retardant laminated bamboo lumber (LBL). The results demonstrated that the flame-retardant treatment using phosphorus–nitrogen–boron compounds endowed the LBL with excellent flame retardancy and smoke suppression properties, even though the bending strength and bond shear strength were slightly reduced. The PF-glued LBL exhibited superior mechanical and shear properties to the MUF-glued ones, primarily due to its higher processing temperature and deeper adhesive penetration. In addition, the MUF-glued flame-retardant LBL displayed better heat release reduction and smoke suppression properties than the PF-glued LBL, which resulted from the synergistic flame retardancy between the melamine element in MUF and the applied flame retardant. The analysis of the influence of adhesive type on the mechanical and fire properties of flame-retardant LBL holds significant importance for the future design and production of high-performance LBL material.

## 1. Introduction

Bamboo is a rapidly growing, sustainable, and renewable resource. Compared with timber, it is lighter, stiffer, and stronger [1]. To enhance the utilization rate of bamboo and improve its consistency, strength, and uniformity, bamboo culm can be split into strips and laminated together using adhesives to produce laminated bamboo lumber (LBL) [2,3]. LBL is characterized by being sturdy, durable, elegant in texture, and environmentally friendly [4]. The extensive applications of LBL in construction, decoration, and furniture have reduced the over-dependence of related industries on wood resources, which is of significance for improving the ecological environment [5].

As bamboo primarily consists of cellulose, hemicellulose, and lignin, its inflammability poses a limitation on its use as a material in construction and decoration [6]. Therefore, it is crucial to treat bamboo with flame retardants to facilitate the use of these engineered bamboo products in structural applications [7]. The most economical and commonly used commercial flame retardants are halogen-containing and phosphorus-based compounds due to their excellent flame retardancy. However, halogen compounds are becoming increasingly limited since they may cause mutagenic and carcinogenic effects. Due to ever-increased attention to the environmental issues associated with halogen-based additives, phosphorus-based flame retardants are gaining higher popularity due to their higher environmental safety and high flame-retarding efficiency [8]. The flame retardancy of the phosphorus–nitrogen compounds, such as monoammonium phosphate (MAP), di-ammonium phosphate (DAP), and ammonium polyphosphate (APP), derives from the combination of the condensed phase and the gas phase for flame retardance [9,10]. During the burning process, these flame retardants decompose into phosphoric acid and poly-phosphoric acid, which form a non-volatile layer of protective film on the surface of the substrate material, thus preventing heat transmission into the material. At the same time, these flame retardants can potentially release ammonia, nitrogen, water vapor, and other non-flammable gases that dilute the combustible gas and reduce the surface temperature of the material by absorbing heat, thus effectively preventing the material from burning [11]. In addition, boron-based flame retardants (boric acid and borax) have some efficacy in retarding flame spread on wooden material surfaces, with low mammalian toxicity and low volatility [12]. In addition to the charring effect, the flame retardants have rather low melting points and could form glassy films when exposed to high temperatures in a fire. Borax tends to reduce flame spread but can promote smoldering or glowing, while boric acid suppresses smoldering but has little effect on flame spread [13]. Therefore, these compounds are normally used together, a combination which also has a good synergistic effect with phosphorus–nitrogen flame retardants [14,15]. The multi-component mixture of boric acid, borax, and phosphorus-based compounds is more effective than one single-element flame retardant, due to the multiple mechanisms of the components in the flame retardant [10,16].

The lamination of bamboo strips to form structural LBL material relies on adequate inter-ply bonding via adhesives. For interior uses and applications with low requirements for weather resistance, melamine urea formaldehyde (MUF) adhesive is generally used [7]. For engineered bamboo products to be seriously considered for exterior applications, phenol formaldehyde (PF) could be a better choice due to its higher durability and water resistance [17]. It is worth noting that the properties of PF adhesive, especially its bonding capability and curing properties, can be affected by flame retardants [18], resulting in deteriorated mechanical properties to the LBL material. However, the adhesive used undergoes thermal decomposition during combustion. This generates volatile substances that are released into the air and condense to form cross-linked polymers on the LBL surface, which influences the combustion process [19,20]. In order to produce high-quality LBL material, it is essential to investigate the relationship between flame retardancy and gluing performance. Improvements in the properties of LBL in terms of dimensional stability and flame retardancy will greatly expand LBL’s applications in structures and building areas. However, to the best of our knowledge, current studies have mainly focused on improving the mechanical properties or fire properties of LBL individually.

In this study, bamboo strips were treated with a flame-retardant mixture consisting of MAP, borax, and boric acid, and then glued with different adhesives (PF and MUF) to make LBL. This study aimed at assessing the impact of flame-retardant compounds on the mechanical properties of treated LBL and examining the flame-retardant properties of LBL using a cone calorimeter. Also, the influence of adhesives on the flame-retardant LBL was investigated. The objective of this study was to gather first-hand data on the relationship between the flame-retardant properties and mechanical properties of LBL material based on different adhesive systems, which provides valuable theoretical support and serves as a reference for the rational development of flame-retardant LBL products, potentially expanding their applications as structural and decorative materials.

## 2. Materials and Methods

### 2.1. Materials

Moso bamboo (*Phyllostachys edulis*) aged 4 years was obtained from Xinchang, Zhejiang province, China. The bamboo culm was split and cut into bamboo strips measuring 1000 mm (longitudinal) × 20 mm (tangential) × 5.5 mm (radial). The bamboo strips were conditioned to the moisture content level of 8–10%. The density of the bamboo strips was 0.75–0.80 g/cm^3^. Phenol formaldehyde (PF) adhesive with a solid content of 47.5% was purchased from Beijing Dynea Chemical Industry Co., Ltd. (Beijing, China). Melamine urea formaldehyde (MUF, solid powder) purchased from Nanjing Chemical Reagent Co., Ltd. (Nanjing, China), was dissolved in DI water to obtain a MUF solution with a solid content of 46.3% before use. Monoammonium phosphate (NH_4_H_2_PO_4_), boric acid (H_3_BO_3_), and borax (Na_2_B_4_O_7_·10H_2_O) were purchased from Sigma Aldrich (St. Louis, MO, USA). All the agents used were commercially available and used directly.

### 2.2. Treatment of Bamboo Strips

A flame-retardant mixture consisting of 70% MAP, 15% boric acid, and 15% borax by weight, according to our previous study [9], was utilized to treat bamboo strips using the vacuum pressure method. The impregnation solution was prepared from this flame-retardant mixture by dissolving it in distilled water at a concentration of 20%. During a typical treatment run, the bamboo strips were placed in a vacuum chamber with a pressure of −0.08 MPa for 30 min, then the flame-retardant solution was sucked into the chamber during the vacuum relief process. Subsequently, the bamboo strips were soaked and 1.0 MPa of pressure was applied and held for 2 h. After the impregnation process was completed, the pressure was released and the bamboo strips were taken out and weighed. The weight gain, determined by calculating the difference in mass before and after the treatment, was 10.3%. Finally, the treated bamboo strips were oven-dried at 60 °C for 12 h, and the final moisture content of the treated bamboo strips was controlled to 8% for subsequent uses.

### 2.3. Preparation of the Flame-Retardant LBL

**Figure 1:**
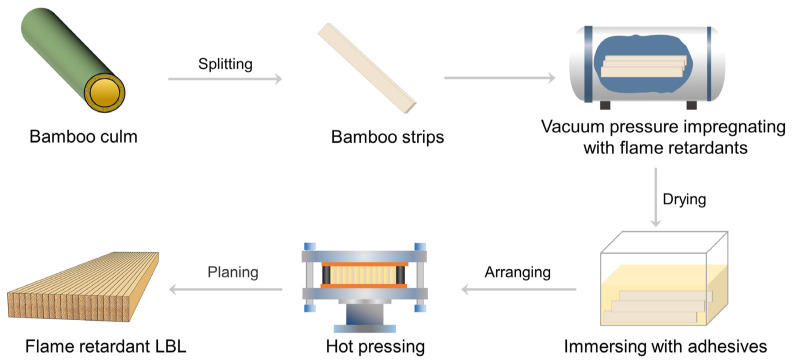
Manufacturing process of flame-retardant LBL.

As illustrated by Figure 1, the treated bamboo strips were immersed in adhesives for 2 h, and then the bamboo strips were laminated and hot pressed with a pressure of 3 MPa. For the PF adhesive, the press temperature and time were 135 °C and 20 min, respectively, while for the MUF adhesive, the press temperature and time were 105 °C and 20 min, respectively. The prepared LBL samples were double-sided planed to result in a thickness of 19 mm. The final flame-retardant LBL samples, glued with PF and MUF, were labeled as LBL-PF-FR and LBL-MUF-FR, respectively. LBL samples glued with PF and MUF without flame-retardant treatment were also prepared for comparison and denoted as LBL-PF and LBL-MUF, respectively. For subsequent tests, all LBL were processed into test samples and conditioned at 65% RH and 20 °C until a constant weight was reached.

### 2.4. Characterization

**Figure 2:**
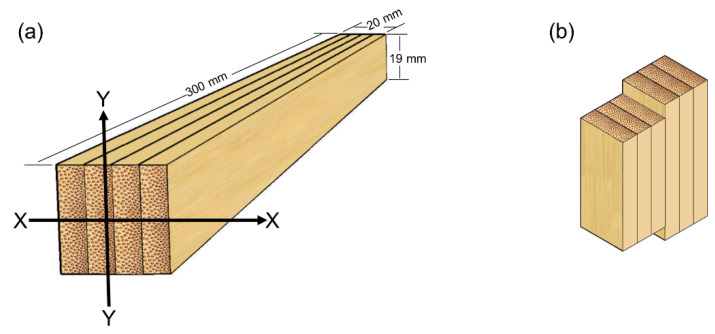
Schematic of (**a**) bending samples and (**b**) shear samples.

A three-point bending test was conducted according to the GB/T 15780-1995 standard [21] using a universal mechanical machine (Instron 5582, Norwood, MA, USA). The dimension of the LBL samples was 300 mm × 20 mm × 19 mm, and a loading speed of 2 mm/min was used. External force was applied along the width direction (X-X) and thickness direction (Y-Y), respectively, as illustrated in Figure 2a. Each test was replicated 10 times.

The step-shear test on the glue line was conducted according to the GB/T 15780-1995 standard on a testing machine (WDW-E100D, Jinan, China) equipped with a load cell capacity of 10 kN, in displacement control at a rate of 0.6 mm/min. The shear plane coincided with the plane of the glue, and the average shear area was 40 × 19 mm. Figure 2b shows that the LBL samples were sheared parallel to the glue surface.

Morphologies of the tested LBL samples were examined using a scanning electron microscope (SEM) on an FEG-XL30 (FEI Company, Eindhoven, NL, USA). All the samples were sputtered with a layer of gold before the SEM analysis.

Cone calorimeter analysis is an effective approach for assessing the combustion behavior of materials. In this study, a cone calorimeter (Fire Testing Technology, East Grinstead, UK) at a heat flux of 50 kW/m^2^ was applied to evaluate the combustion performance of the flame-retardant LBL samples. The bottom and sides of the squared LBL samples (100 mm × 100 mm × 19 mm) were wrapped with aluminum foil prior to the test. Key properties measured in this test included heat release rate (HRR, kW/m^2^), total heat release (THR, MJ/m^2^), mass (%), mass loss rate (MLR, g/s), total smoke production (TSP, m^2^), and smoke production rate (SPR, m^2^/s).

## 3. Results and Discussion

### 3.1. Mechanical Properties

**Figure 3:**
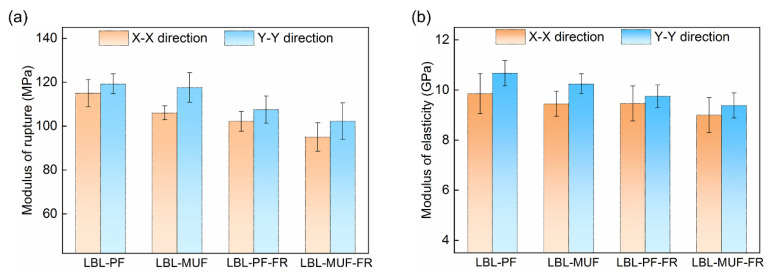
Mechanical properties of LBL samples. (**a**) modulus of rupture; (**b**) modulus of elasticity.

According to Figure 3, the average modulus of rupture (MOR) and modulus of elasticity (MOE) of LBL in the Y-Y direction were considerably higher than those in the X-X direction, which was attributed to the gradient structure of bamboo in the radial direction [22]. After the flame-retardant treatment, the treated LBL samples exhibited decreased bending strength compared to the untreated groups, with an average MOR decrease of 10.3–13.1% and an average MOE decrease of 3.9–8.7%. The LBL made from PF showed higher MOR and MOE than the MUF-glued LBL regardless of whether flame-retardant treatment was used or not. This can be explained by the fact that PF-glued LBL preparation involved a high temperature for hot pressing, allowing the adhesives to penetrate into the interior of the bamboo, resulting in better solidification and bonding. Additionally, the boric acid in the composite flame retardant increased the toughness of the PF adhesive and improved its bonding strength [23].

**Figure 4:**
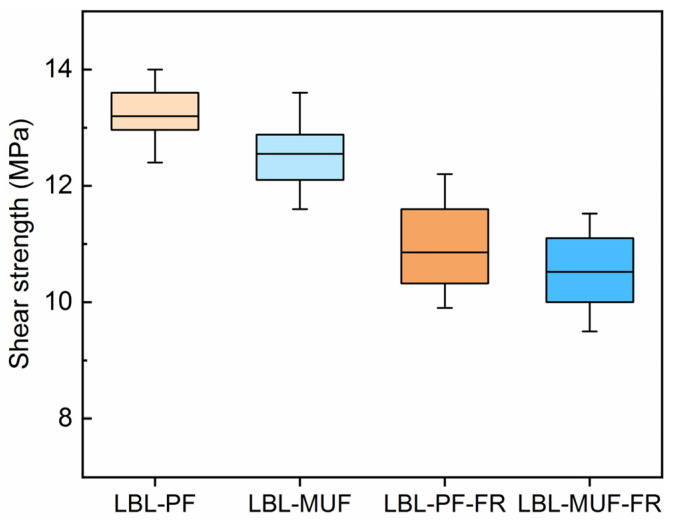
Shear strength of LBL samples.

To use bamboo as a structural material, the bonding properties must satisfy some specific standard requirements. The shear strength of LBL can be a proper indicator of the bond performance between the bamboo strips (Figure 4). After flame-retardant treatment, the shear strengths of LBL-PF-FR and LBL-MUF-FR were 10.85 MPa and 10.52 MPa, respectively, representing reductions of 17.8% and 16.2% compared to the LBL-PF and LBL-MUF. These reductions can be attributed to the acidic nature of flame-retardant components, as boric acid and MAP are both acidic, which can shorten the gelation time for PF [24]. The shear strength of LBL glued with PF and MUF exhibited a similar trend to the bending properties. Specifically, the shear strength of the LBL prepared with PF was superior to that prepared with MUF. The curing properties of an adhesive highly depend on the surface conditions. In the case of the phosphorus–nitrogen flame retardant with water-soluble groups, the chemicals that precipitate on the bamboo surface after the flame-retardant treatment might affect the gluing efficacy, which could also result in a fast curing rate for MUF [25]. Although these commonly used industrial flame retardants are water-soluble and have a certain degree of hygroscopicity, they have been heavily commercialized in the industry because of their excellent flame retardant efficiency and cost effectiveness. Weathering tests will also be required to determine the depletion of boron and phosphorus compounds in exterior conditions. Further investigation will also be carried out for developing leach-resistant flame retardants with economic potential.

### 3.2. SEM Analysis

**Figure 5:**
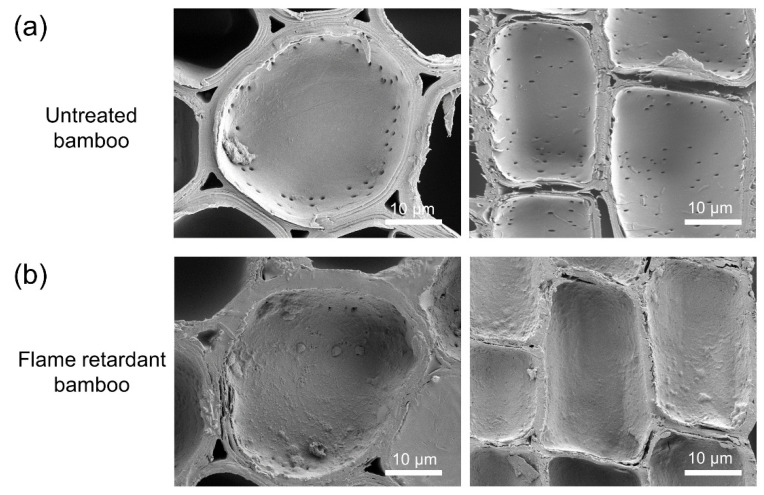
SEM images of (**a**) untreated bamboo and (**b**) flame-retardant bamboo.

Figure 5 presents the SEM images of bamboo samples. It is obvious that the surface of the untreated bamboo cell lumen (parenchyma cell) was smooth, with clearly visible pits. After treatment with the flame retardant, a dense flame-retardant coating covered the surface of the bamboo cell lumen and the pits were filled up, suggesting that the flame retardant had penetrated into the bamboo cell lumen through vacuum pressure impregnation treatment. The penetration of the flame retardant into the cell walls is closely related to the improvement in flame retardancy and smoke suppression [26]. Prior research claimed that parenchyma cells have thinner cell walls and lots of pits, which are beneficial for causing deeper adhesive penetration [27]. However, the presence of an attached flame-retardant coating on the cell lumen filled up these pits, which hindered the direct and effective contact between the bamboo strips and adhesives on the bonding interfaces, leading to reduced bonding strength and bending properties. This was unavoidable due to the need for generating sufficient flame retardancy with high loading of the flame retardant. It is still possible to compensate for the reduction with other potential avenues to improve the bonding properties.

### 3.3. Influence of PF and MUF on Fire Properties

**Figure 6:**
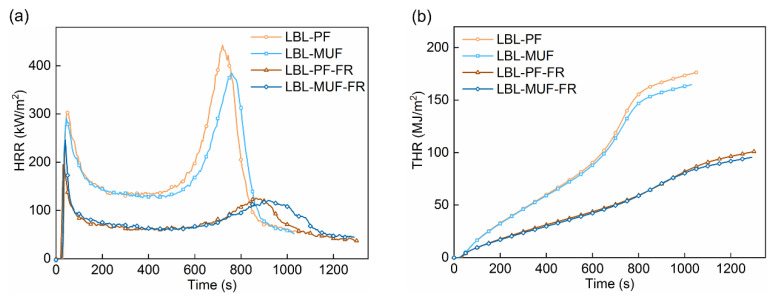
HRR (**a**) and THR (**b**) curves of LBL samples.

The cone calorimeter test was used to assess the combustion behavior of LBL based on the oxygen consumption principle. In Figure 6 and Table 1, it can be observed that all LBL samples exhibited two sharp HRR peaks, which are due to the special characteristic of char-forming substances on wood-based materials [28]. Then, a drop to a steady state of heat release rate was observed, followed by a second peak as the final portion of LBL was consumed. The higher peak heat release rate (PHRR) can be directly attributed to the great amount of oxygen consumption during combustion, resulting in higher generation of heat energy in a fully developed fire. The second HRR peaks (PHRR_2_) of LBL-PF-FR and LBL-MUF-FR were 125.58 kW/m^2^ and 120.80 kW/m^2^, which decreased by 71.6% and 68.5% separately compared to the LBL-PF and LBL-MUF, and the corresponding appearance times were delayed by 145 s and 160 s, respectively. These findings suggested that the flame-retardant treatment can effectively inhibit the final deflagration and exothermic reaction of LBL when exposed to a fire, thus improving the fire resistance of LBL.

In addition, the THR of LBL-PF-FR and LBL-MUF-FR remarkably decreased by 42.7% and 42.0%, respectively, compared to the untreated LBL, indicating that the treated LBL had excellent flame retardancy and exhibited lower heat load in the fire. Regardless of whether the flame-retardant treatment was used or not, the PHRR and THR of LBL glued with MUF were slightly lower than those of LBL glued with PF. This can be attributed to the presence of the N element in the MUF adhesive, which has a synergistic flame-retardant effect with the P element in the applied flame retardant [29]. Additionally, the presence of hydroxyl and methylene groups in PF adhesive made the glued LBL susceptible to oxidation, resulting in less thermal stability and a certain impact on flame retardancy [30].

**Figure 7:**
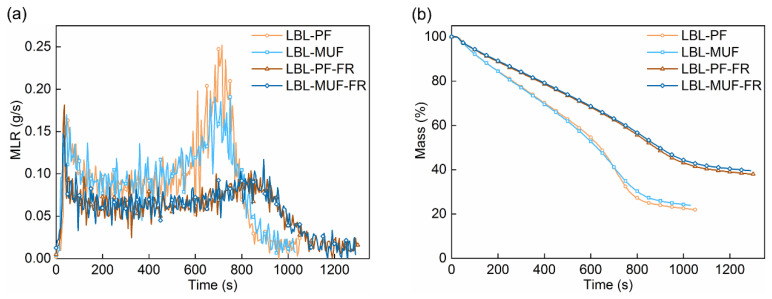
MLR (**a**) and Mass (**b**) curves of LBL samples.

MLR refers to the material mass change during combustion. Figure 7 shows the MLR and Mass curves of LBL. The MLR curves of LBL displayed a similar trend to their HRR curves, indicating that mass loss and heat release occurred simultaneously during combustion. In the initial stage of combustion, when the char layer on LBL surfaces was not formed, there was a higher rate of mass loss, represented by the first peak on the MLR curves. In the later stage of combustion, LBL experienced a significant mass loss due to the decomposition and rupture of the remaining material, as demonstrated by the second peak on the MLR curves.

A similar trend was observed when comparing the Mass curves of all LBL samples. During the flaming phase, the mass of the residual material declined rapidly. The application of the flame retardant significantly improved the thermal stability of LBL, as indicated by the smaller slope of the Mass curves. This was associated with a decreased pyrogenic decomposition rate. In the final stage, the Mass curves turned almost horizontal, indicating the calcination of char residue. Figure 7 and Table 1 show that the treated LBL had a relatively small mass loss and mass loss rate, resulting in a relatively higher mass residue percentage of 38.12% (LBL-PF-FR) and 39.47% (LBL-MUF-FR), respectively, while for LBL-PF and LBL-MUF, the mass residue percentage were 21.93% and 23.94%. The formation of char on the LBL’s surface was markedly elevated by the flame-retardant compounds, which played a vital role in reducing the mass loss rate. LBL-PF displayed a higher mass loss and a subsequently lower mass residue percentage than LBL-MUF. This can be attributed to the higher amount of heat released from the combustion of LBL-PF, resulting in greater material degradation.

**Figure 8:**
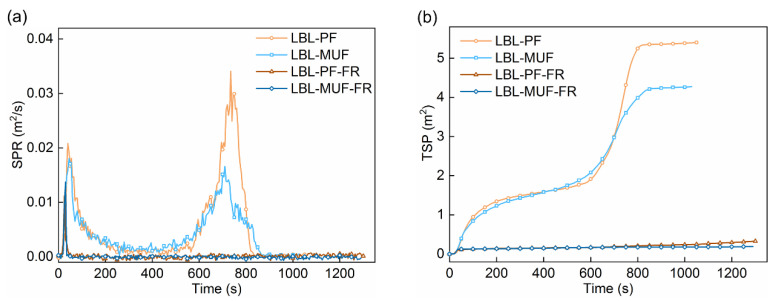
SPR (**a**) and TSP (**b**) curves of LBL samples.

Most fire victims die from excessive inhalation of smoke, which mainly consists of a very large number of toxic components [31]. Therefore, assessing the fire safety of materials also involves considering their smoke production. As shown in Figure 8, the SPR curves can be interpreted as showing that smoke was primarily generated in the early and later stages of combustion, and the trend of SPR was similar to the HRR. This is likely due to the fact that the char formed at low temperatures was oxidized and dissociated at higher temperatures in the later stage, resulting in the release of heat that was transmitted to the inner material and the oxygen-deficient combustion of residual mass produced more smoke [32]. The first SPR peaks for the flame-retardant LBL were lower than those of the untreated LBL, and the second SPR peaks for LBL-PF-FR and LBL-MUF-FR almost disappeared.

The final TSP of LBL-PF-FR and LBL-MUF-FR were 0.33 m^2^ and 0.19 m^2^, respectively, which were 93.9% and 95.6% lower than those of LBL-PF and LBL-MUF. This indicated a superior smoke suppression effect of the applied flame retardant. The generation of N_2_ and NH_3_ gas from the decomposition of the flame retardant could dilute the flammable gases generated from bamboo’s thermal degradation, and then reduced the heat release and smoke release during combustion. Additionally, the presence of borate promoted the carbonization of bamboo, and the formation of a glassy film hindered heat diffusion into LBL and delayed the evaporation of flammable gases [12], which eventually inhibited LBL combustion and smoke release.

The analysis of TSP also showed that LBL glued with MUF was more effective in smoke suppression, a similar trend to the heat release and mass loss rate. This might be attributed to the tight internal structure of the PF-glued LBL, which led to insufficient combustion in the later stage and generated a higher amount of smoke. In contrast, the melamine element in MUF exhibited a certain synergistic effect with the P-N-B-containing flame retardants on retarding fire and smoke. Meanwhile, the pyrolysis of the MUF adhesive could release N_2_ and other noncombustible gases, which reduced the concentration of combustible gases produced by bamboo’s thermal decomposition. Thus, the rate of the gas-phase oxidation reaction was slowed down, ultimately reducing smoke release [33].

## 4. Conclusions

In this study, the mechanical and fire properties of flame-retardant LBL bonded with PF and MUF adhesives were investigated. Impregnation with a phosphorus–nitrogen–boron flame-retardant mixture endowed LBL with excellent flame retardancy and smoke suppression properties. Due to the attached flame-retardant coating on the cell lumen, effective contact between the bamboo strips and adhesives on the bonding surfaces was hindered somehow, which contributed to a slightly reduced bending strength and bond shear strength in the flame-retardant LBL. In addition, PF-glued LBL exhibited better mechanical and shear properties than MUF-glued LBL due to the higher processing temperature for PF and its deeper adhesive penetration. The MUF-glued flame-retardant LBL showed less heat release and better smoke suppression properties than the PF-glued flame-retardant LBL because of the synergistic flame retardancy of the melamine element in MUF with the P-N-B-containing flame retardants. Overall, the analysis of the influence of adhesive types on the mechanical and fire properties of LBL provides valuable insights for future design and production of high-performance LBL material.

## Figures and Tables

**Table 1:** Cone calorimeter results for LBL samples.

Sample	PHRR_1_ (kW/m^2^)	PHRR_2_ (kW/m^2^)	THR (MJ/m^2^)	Average MLR (g/s)	Mass (%)	TSP (m^2^)	PSPR (m^2^/s)
LBL-PF	302.58	442.63 (720 s)	176.35	0.086	21.93	5.40	0.034
LBL-MUF	291.76	383.92 (760 s)	164.73	0.081	23.94	4.27	0.017
LBL-PF-FR	195.68	125.58 (865 s)	101.03	0.059	38.12	0.33	-
LBL-MUF-FR	246.25	120.80 (920 s)	95.50	0.057	39.47	0.19	-

## Data Availability

Supplemental data can be provided upon reasonable request.
